# New Vaccine Formulations Containing a Modified Version of the Amastigote 2 Antigen and the Non-Virulent *Trypanosoma cruzi* CL-14 Strain Are Highly Antigenic and Protective against *Leishmania infantum* Challenge

**DOI:** 10.3389/fimmu.2018.00465

**Published:** 2018-03-15

**Authors:** Ana Paula M. M. Almeida, Leopoldo F. M. Machado, Daniel Doro, Frederico C. Nascimento, Leonardo Damasceno, Ricardo Tostes Gazzinelli, Ana Paula Fernandes, Caroline Junqueira

**Affiliations:** ^1^Departamento de Análises Clínicas e Toxicológicas, Faculdade de Farmácia, Universidade Federal de Minas Gerais, Belo Horizonte, Brazil; ^2^Instituto René Rachou, Fundação Oswaldo Cruz, Belo Horizonte, Brazil; ^3^Hertape Saúde Animal S.A., Ceva Saúde Animal Ltda, Juatuba, Brazil; ^4^Division of Infectious Disease, University of Massachusetts Medical School, Worcester, MA, United States

**Keywords:** visceral leishmaniasis, vaccine, *Trypanosoma cruzi* CL-14, *Leishmania infantum*, amastigote 2

## Abstract

Visceral leishmaniasis (VL) is a major public health issue reported as the second illness in mortality among all tropical diseases. Clinical trials have shown that protection against VL is associated with robust T cell responses, especially those producing IFN-γ. The *Leishmania* amastigote 2 (A2) protein has been repeatedly described as immunogenic and protective against VL in different animal models; it is recognized by human T cells, and it is also commercially available in a vaccine formulation containing saponin against canine VL. Moving toward a more appropriate formulation for human vaccination, here, we tested a new optimized version of the recombinant protein (rA2), designed for *Escherichia coli* expression, in combination with adjuvants that have been approved for human use. Moreover, aiming at improving the cellular immune response triggered by rA2, we generated a recombinant live vaccine vector using *Trypanosoma cruzi* CL-14 non-virulent strain, named CL-14 A2. Mice immunized with respective rA2, adsorbed in Alum/CpG B297, a TLR9 agonist recognized by mice and human homologs, or with the recombinant CL-14 A2 parasites through homologous prime-boost protocol, were evaluated for antigen-specific immune responses and protection against *Leishmania infantum* promastigote challenge. Immunization with the new rA2/Alum/CpG formulations and CL-14 A2 transgenic vectors elicited stronger cellular immune responses than control groups, as shown by increased levels of IFN-γ, conferring protection against *L. infantum* challenge. Interestingly, the use of the wild-type CL-14 alone was enough to boost immunity and confer protection, confirming the previously reported immunogenic potential of this strain. Together, these results support the success of both the newly designed rA2 antigen and the ability of *T. cruzi* CL-14 to induce strong T cell-mediated immune responses against VL in animal models when used as a live vaccine vector. In conclusion, the vaccination strategies explored here reveal promising alternatives for the development of new rA2 vaccine formulations to be translated human clinical trials.

## Introduction

Visceral leishmaniasis (VL) is an infectious disease that affects mainly splenic and hepatic systems, being the second among the parasitic infections with highest mortality rates. This vector-borne tropical infection is caused by a flagellate protozoan of the genus *Leishmania spp* and affects more than 200,000 people per year (1). Failure to develop a potent human vaccine against this pathogen lies in the complexity of the immune response to the intracellular stage of parasite, given its previously reported defense mechanisms against innate and adaptive immunity (2).

The induction of a strong and long-lasting T helper type 1 (Th1) cellular immune response with high levels of IFN-γ production is a key feature, desired in ideal vaccine candidates. IFN-γ knockout mice fail to overcome infection with *Leishmania* species to which most immunocompetent mouse lines are resistant (3). In addition, IFN-γ is known to induce macrophage activation by increasing nitric oxide (NO) synthesis, leading to NO-mediated killing of intracellular pathogens (4).

The amastigote 2 (A2) proteins are mostly composed of a sequence of 10 amino acids that is repeated 40–90 times with molecular weights varying from 45 to 100 kDa (5). The A2 protein plays an important role in *Leishmania* survival in visceral organs, since the growth of A2-deficient amastigotes of *Leishmania donovani* into visceral organs was severely impaired when compared to A2-containing amastigotes (6). Similarly, the introduction of the *L. donovani* A2 gene into *Leishmania major* enhanced the ability of the latter to survive in visceral organs (7).

The recombinant A2 protein is recognized by human T cells (8) and has been reported as a vaccine candidate in different strains of mice using different vaccine formulations and delivery methods (9–11). In association with saponin, it was shown to induce protection against VL in beagle dogs (12). In all of these studies, protection was accompanied by a strong Th1 immune response with high levels of IFN-γ. In addition, successful vaccination against canine VL has been achieved in the previous years with the administration of Leish-Tec^®^, a commercial vaccine formulated with A2 (13, 14). However, due to its repeat-rich sequence, the expression and purification of recombinant A2 can be a laborious and time-consuming process. In this study, we optimized the A2 gene sequence for expression in *Escherichia coli*, including the entire non-repetitive domain of the protein and 10 repeated regions, in contrast with 40–90 repetitions from the original sequence.

Live vaccine vectors have been shown to improve T cell immune responses elicited by different antigens against several parasitic diseases (15, 16), including the A2 antigen (10). The *Trypanosoma cruzi* CL-14 non-virulent strain has been previously shown to be highly immunogenic in mouse models with production of high levels of IFN-γ and transient expansion of splenic CD8^+^ T cells (17, 18). Moreover, a single injection of CL-14 has been shown to induce protection against lethal *T. cruzi* challenge using the highly virulent Y strain in BALB/c mice (19). To date, no studies have addressed whether immunization with CL-14 is enough to induce protection against VL challenge although both pathogens share a vast proteomic core and similar biology (20, 21). A previous study from our group tested the CL-14 strain as a vaccine vector, becoming a promising antigen delivery strategy to boost Th1 antigen-specific immune response (18).

To further explore the potential of A2 as a candidate antigen for human VL vaccines, we tested an optimized rA2 in combination with adjuvants approved for human vaccination. In addition, we generated stably transfected *T. cruzi* CL-14 expressing A2. Our data suggest that the newly designed rA2, formulated either with monophosphoryl lipid A (MPLA) or the synthetic oligodeoxynucleotides (ODNs) CpG B297, as well as the live vector *T. cruzi* CL-14 vaccine, can elicit robust antileishmaniasis immune responses. While the recombinant protein formulation has proven to be a good candidate to progress to human clinical trials, the live CL-14 vector vaccine may be a future alternative to achieve the prophylaxis of leishmaniasis and Chagas disease.

## Materials and Methods

### Parasites

Epimastigote forms of non-pathogenic *T. cruzi* CL-14 were cultured in liver infusion tryptose medium supplemented with 10% fetal bovine serum (FBS) (Gibco, USA), 10,000 U/mL penicillin, and 10 mg/mL streptomycin (Gibco, USA) at 28°C in a biochemical oxygen demand (BOD) incubator. The transgenic parasites obtained were cultured in the same conditions with addition of 250 µg/mL geneticin (Gibco, USA) for selection of neomycin-resistant parasites. To perform immunization protocols, parasites were kept in stationary culture for 15–20 days, aiming to obtain metacyclic forms. Promastigotes of *Leishmania infantum* (MOM/BR/1970/BH46) were cultured in Schneider’s Insect medium (Sigma Aldrich, USA) supplemented with 10% FBS, 10,000 U/mL penicillin, and 10 mg/mL streptomycin at 26°C in a BOD incubator.

### Mice and Ethics Statement

Female BALB/c mice, 6- to 8-week-old mice were purchased from the Institute of Biological Sciences at Federal University of Minas Gerais (Belo Horizonte, Brazil). Mouse experiments were approved by and conducted according to animal welfare guidelines of the Ethics Committee of Animal Experimentation from Federal University of Minas Gerais, under the approved protocol number 73/2009.

### Antigen Expression and Purification

To improve the A2 protein expression and purification protocols, without impairing the immunological outcome generated by this antigen, we constructed an *E. coli* codon-optimized gene containing a selected nucleotide sequence spanning the entire coding region of the A2 protein. This improved gene contains 10, instead of the 40–90 repeated regions present in the original *L. infantum* A2 gene (XM_001465551) (22) and additional codons for a *C*-terminal 6× His tag. The synthetic gene was further cloned, through restriction enzyme digestion, into the pET9a vector to originate the plasmid pET9a24a-A2His, which was then transformed into *E. coli* C41 cells.

Protein expression was induced by IPTG. After induction, cells were harvested by centrifugation and lysed by sonication. The supernatant was then used for purification under denaturation conditions in a HisTrap column (GE Healthcare, UK) using the Äkta prime system. The purified protein was submitted to a second purification step using a HiTrap™Sepharose^TM^ HP Ion Exchange column (GE Healthcare, UK). Finally, the purified fraction was loaded in an EndoTrap^®^ HD column (Hyglos, DE) to remove endotoxins. The final purified protein was analyzed in Coomassie-stained SDS-PAGE, and its specific reactivity was assessed by western blot analysis using an anti-A2 monoclonal antibody, kindly provided by Dr. Greg Matlashewski.

### Parasite Construction and Characterization

The codon-optimized A2 gene was isolated and cloned into the pROCKNeo plasmid using the *XhoI* and *XbaI* restriction sites. The resulting plasmid, pROCKNeoA2His (Figure S1 in Supplementary Material) was then used to transfect *T. cruzi* epimastigotes, as previously described (23). To achieve an integrative transfection into the parasite genome, the plasmids were linearized with *Not*I prior to electroporation. After transfection, the parasites were selected for neomycin resistance by addition of geneticin (G418) into culture medium. The presence of the A2 gene into the selected parasites was assessed by conventional PCR using: forward primer: HX1-5′ TTCTTCAAAATATGCAGCGGATT 3′ and reverse primer for A2: A2NOTRV-5′ TACCGCGGCCGCCTAGTGGTGATGG 3′.

Expression of the A2 protein in the recombinant *T. cruzi* CL-14 A2 parasite was assessed by both western blot and immunofluorescence analysis. For western blot analyses, the cell lysates of 2 × 10^7^ epimastigotes of *T. cruzi* CL-14 or *T. cruzi* CL-14 A2 were submitted to SDS-PAGE and then transferred to a 0.45-μm nitrocellulose membrane (GE Healthcare, UK). To detect A2 expression by the *T. cruzi* CL-14 A2, we used a 1:100 dilution of anti-A2 monoclonal antibody. As the secondary antibody, we used anti-mouse anti-IgG horseradish peroxidase-conjugated antibody (Sigma Aldrich USA). The chemiluminescence reagent ECL Prime (GE Healthcare, UK) was used to reveal the reactions. The membranes were analyzed by an IS600 image system (GE Healthcare, UK).

For immunofluorescence analysis, 2 × 10^5^ parasites were washed, fixed [phosphate-buffered saline (PBS) + 2% paraformaldehyde], loaded into a poly-l-lysine-coated glass slide, and incubated overnight at room temperature. The cells were then rehydrated and permeabilized with Triton X-100 0.2% in PBS. The monoclonal anti-A2 antibody was used as a primary antibody. Secondary antibody Alexa Fluor^®^ 488 anti-mouse IgG (Thermo Scientific, USA) was used. After five wash steps, we added Vectashield containing DAPI to each spot (Vector Laboratories, USA). The glass slides were analyzed in a LSM Zeiss microscope.

### Immunization Protocols and Challenge

For the immunization protocols, BALB/c mice (*n* = 10) were vaccinated with homologous prime-boost protocols. For the immunization groups receiving the recombinant protein, the following formulations were used: 10 µg of rA2 associated with 18 µg of CpG B297 (24) adsorbed in 30% (v/v) of Alum Rehydragel LV solution (Reheis, USA) or 10 µg of rA2 associated with 1.0 µg of MPLA (Butantan, Brazil). A total volume of 100 μL/mice was used as the dose. The negative control group received PBS. Both, protein and control group immunizations were done subcutaneously at the base of the tail, while the live vaccine groups received either 10^7^ metacyclic forms of the CL-14 A2 or the CL-14 wild-type parasite, administered by intraperitoneal injections. For all protocols, the first dose was administered on the day 0, and each group was boosted 4 weeks later, with the same immunization dose.

For assessment of immunogenicity, mice (*n* = 4) were euthanized 21 days after the last vaccination dose. Serum samples and spleen of all groups were collected and used to assess, respectively, B and T cell immune responses elicited by the immunization protocols. Immunized mice (*n* = 6) were then infected subcutaneously, 21 days after the last vaccination dose, into the right hind footpad, with 1 × 10^7^ stationary phase promastigotes of *L. infantum*. After challenge (30 days), the animals were euthanized; whole spleen was collected, individually processed, and used for parasite burden estimation. All experiments were performed twice, and representative results of each analysis are shown.

### Immunological Assays

For IFN-γ and IL-10 production assays, 1 × 10^6^ splenocytes from vaccinated mice were incubated at 37°C and 5% CO_2_ for 48 h in the presence of 10 µg/mL rA2 or RPMI (non-stimulated control). The IFN-γ concentrations in the cell culture supernatants were determined using OptEIA™ Mouse IFN-γ ELISA Kit (BD, USA), according to manufacturer’s instructions. OptEIA™ Mouse IL-10 ELISA Kit (BD, USA) was used to evaluate IL-10 concentration, according to manufacturer’s instructions.

Antibody production was measured by enzyme-linked immunosorbent assay (ELISA), using rA2 protein as the coating antigen, at the concentration of 10 µg/mL. Serum from each immunized mouse was diluted 1:50, as established by a previous titration curve, and analyzed in duplicate. The reaction was developed using horseradish peroxidase-conjugated secondary antibodies (anti-IgG, IgG1, and IgG2a) (Zymed, USA) and substrate solution (Ortho-phenylenediamine + H_2_O_2_ diluted in citrate buffer pH 5,0). Optical densities were measured at 492 nm in the VersaMax microplate reader (Molecular Devices, USA).

To guarantee that effective immunization using *T. cruzi* CL-14 vector were achieved, we also evaluated the antibody response directed to the live vaccine vector by ELISA. For this purpose, total *T. cruzi* CL-14 extract was used as the coating antigen at a concentration of 10 µg/mL, and the reaction was developed as described above.

### Estimation of Parasite Load

For detection of *L. infantum* infection, 30 days after the challenge, animals were euthanized, and their spleens were harvested for parasite quantification by qPCR. DNA extraction of the analyzed organs was performed using the Illustra tissue and cells Mini Spin Kit (GE Healthcare, UK) according to the manufacturer’s instructions. After extraction, samples were quantified in a NanoVue spectrophotometer (GE Healthcare, UK) and diluted with nuclease-free water to a final concentration of 10 ng/µL.

SYBR Green qPCR was performed, as previously described (25), with primers targeting the sequences of *L. infantum* KDNA (accession number: EU437407), namely, KDNA fw: 5’ CCT ATT TTA CAC CAA CCC CCA GT 3’ and kDNA Rv: 5’ GGG TAG GGG CGT TCT GCG AAA 3’ and also *Mus musculus* β actin gene (accession number: NM_007393.5) namely, actin fw: 5’ CAG AGC AAG AGA GGC ATC C 3’ and actin Rv: 5’ TCA TTG TAG AAG GTG TGG TGC 3’. The qPCR amplification resulted in a 116-bp product for the kDNA primers and a 104-bp product for the actin β primers. Reactions were performed in a final volume of 20 µL, consisting of 1× MAXIMA SYBR Green/ROX qPCR Master Mix (Thermo Scientific, USA), 4 nM of each primer, and 50 ng of DNA template.

Standard curves were performed using eight serial dilutions of the target DNA, starting from 500,000–0.05 pg. All samples and negative controls were analyzed in duplicates for each run. Parasite loads were estimated as the number of *Leishmania* cells in 10^6^ mouse cells. For determination of percentage of protection, the parasite loads in control PBS mice were considered as 100%. Reductions in parasite loads in vaccinated mice were then converted to percentage values.

### Sequences Comparison

Sequence similarities between optimized A2 and proteins belonging to *T. cruzi* were accessed using BLASTp (NCBI, USA) analysis specific to *T. cruzi* strain CL Brener (taxid:353153) databank.

### Statistical Analyses

Statistical analyses were performed using GraphPad Prism 7.0 (GraphPad, CA, USA). For data analysis and comparisons, we used one-way ANOVA followed by Tukey’s multiple comparisons posttest. *p* < 0.05 was considered statistically significant (*p < 0.05, **p < 0.01, and ***p < 0.001,).

## Results

### Immunogenicity and Protection Conferred by rA2 Protein

**Figure 1:**
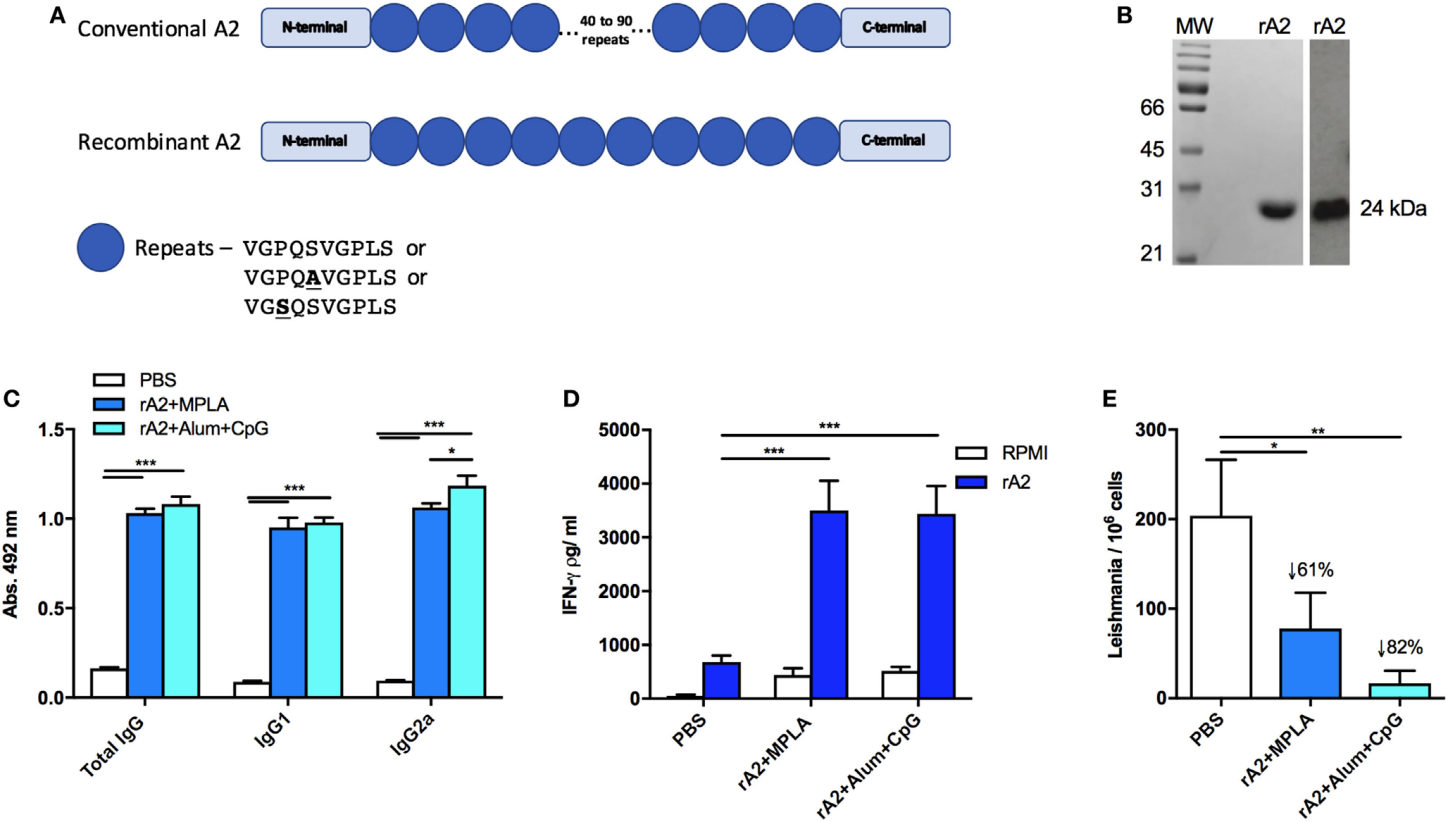
Recombinant amastigote 2 (A2) confers immunogenicity and protection against *Leishmania infantum* challenge in BALB/c mice. **(A)** Schematic representation of the recombinant A2 protein compared with the original A2 sequence present in *Leishmania donovani*. Boxes represent the *C*- or *N*-terminal sequences, while the circles represent the repetitive amino acid sequences. Although the codon optimization was performed, it is not represented here. **(B)** Coomassie-stained SDS-PAGE of purified rA2 (left panel) and specific reactivity in Western blot with an anti-A2 monoclonal antibody (right panel). **(C)** Mice immunized s.c. with a homologous prime-boost protocol containing phosphate-buffered saline (PBS; negative control) or 10 µg of rA2 administered along monophosphoryl lipid A (MPLA) or Alum/CpG had their serum obtained 21 days after the last immunization dose. Serum samples were individually analyzed by enzyme-linked immunosorbent assay (ELISA) for total IgG, IgG1, or IgG2a, as previously described. **(D)** Mice immunized as previously described were sacrificed 21 days after the last immunization, and spleen cells were used for IFN-γ analyses by ELISA. For *in vitro* restimulation, it was used either rA2 or RPMI medium. **(E)** Mice immunized as previously described were euthanized 30 days after the challenge with *L. infantum*, and spleens were used for parasite loads’ determination using qPCR assay. *p < 0.05, **p < 0.01, and ***p < 0.001 analyzed by one-way ANOVA followed by Tukey’s multiple comparison test. Results are expressed as the mean and SEM values of duplicate assays using four to six different animals from each group individually analyzed.

The recombinant A2 antigen has been employed in several preclinical studies against leishmaniasis (9, 26) and is also commercially available as a canine vaccine against VL (13, 27). In this study, we developed an optimized A2 gene for expression in *E. coli*, including the entire non-repetitive domain of the protein and only 10 repeated regions, in contrast to 40–90 repetitions from the original sequence (Figure 1A). Therefore, this sequence has become more suitable for bacterial expression and should maintain the same immunogenicity as the original sequence. The protein obtained from *E. coli* expression followed by affinity purification is shown in the Coomassie-stained SDS-PAGE (left panel), while the anti-A2 specificity was confirmed by western blot analysis (right panel), both showing a band around 25 kDa, as expected for the optimized protein (Figure 1B).

To confirm the immunogenicity elicited by the optimized rA2, immunization protocols were performed as follows: BALB/c mice were immunized with PBS (control group) or rA2 in combination either with MPLA or CpG B297 plus Alum as adjuvant. To assess the humoral immune response, serum from immunized mice was subjected to ELISA anti-rA2 for IgG total, IgG1, and IgG2a antibody isotypes (Figure 1C). The data show that the rA2 protein, associated with either MPLA or CpG B297 adjuvants, was able to elicit a robust IgG total, IgG1, and IgG2a production, as previously observed with the original sequence (26).

Previous studies reporting vaccination strategies against *Leishmania* infection disclosed the importance of cell-mediated immunity for protection; specifically, the cellular responses based on the IFN-γ production. Therefore, by way of an ELISA assay, we measured the levels of IFN-γ present in the supernatants of splenocyte cultures from immunized mice after *in vitro* stimulation with rA2 or RPMI for 48 h. Results showed that the immunization with the rA2 antigen associated with MPLA or Alum/CpG led to high levels of IFN-γ (Figure 1D), when compared with PBS group under rA2 stimuli.

After the ability of the rA2-based vaccines to stimulate a Th1-biased T cell response had been confirmed, we tested if such immune response could induce protection against challenge with *L. infantum*. For this, parasite burden was evaluated in the spleen of mice in each immunization group 30 days after the challenge with 1 × 10^7^ *L. infantum*. The results showed that the optimized rA2 antigen was capable of inducing a strong reduction of the parasite burden regardless of the adjuvant used (Figure 1E). However, these results support the potential use of the optimized rA2 antigen as a vaccine candidate for VL.

### Stably Transfected CL-14 Parasites Express Recombinant A2 Proteins

**Figure 2:**
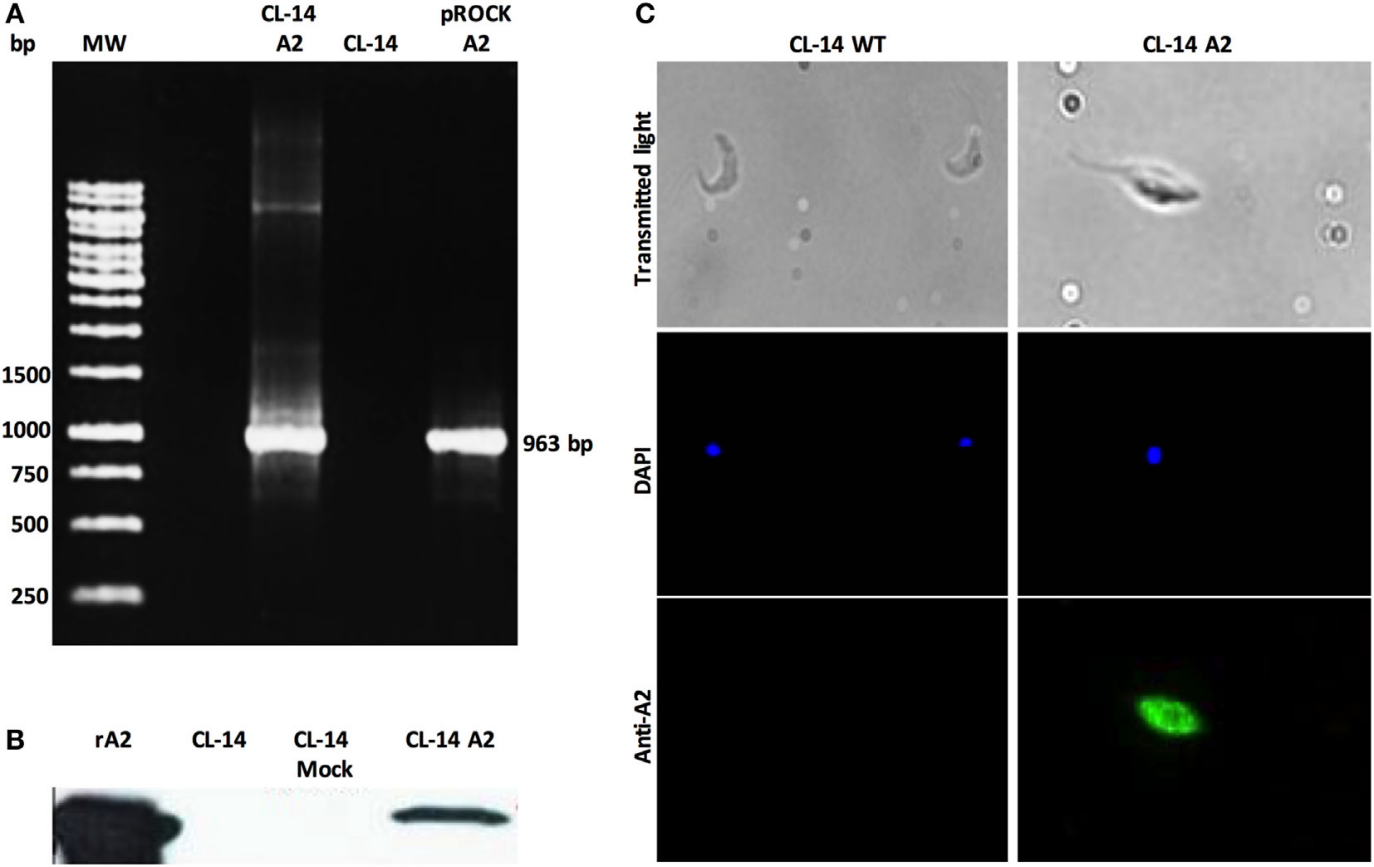
Transgenic CL-14 parasites stably express recombinant amastigote 2 (A2). **(A)** Agarose gel electrophoresis of PCR products confirming specific amplification of the nucleotide sequences enconding the A2 (963pb) antigen by CL-14 A2 DNA extracts. As a positive control, plasmid DNA containing the nucleotide sequence-encoding rA2 was used (see Supplementary Material for plasmid maps). For negative control, we used untransfected CL-14 DNA extract. **(B)** Western blot analysis of transgenic CL-14 parasites lysates confirming the expression of 24 kDa A2. We used rA2 as a positive control. Monoclonal anti-A2 was used for detection of A2. **(C)** Immunofluorescence of fixed untransfected (CL-14 WT) and trasngenic CL-14 A2 parasites. 2 × 10^5^ parasites were washed, fixed, and loaded into a poly-l-lysine-coated glass slide. DAPI shows nuclear staining, and the green channel shows protein localization detected by monoclonal anti-A2 with AlexaFluor 488 anti-mouse IgG as secondary antibody. The glass slides were analyzed in a LSM Zeiss microscope.

Aiming to obtain transgenic *T. cruzi* CL-14 lines, we engineered an integrative plasmid with the vector pROCKNeo containing the coding sequence for A2 (Figure S1 in Supplementary Material). The pROCKNeo backbone contains sequences of homology to the abundantly prevalent locus of *T. cruzi* β-*tubulin* gene, allowing insertion of a multi-copy transgene into the genome of the parasite (23). Successful transfection of the recombinant plasmid into the parasite was confirmed *via* PCR using oligonucleotides flanking the A2 coding sequence. PCR results indicate that the constructs containing the A2 gene were efficiently inserted into (Figure 2A). Plasmids containing the construct (pROCKNeoA2) and the genomic DNA, derived from wild-type CL-14, were used, respectively, as positive and negative controls.

To confirm whether the neomycin-resistant CL-14 transfected parasites were expressing the recombinant protein, we used parasite extracts in western blot analysis. Recombinant A2 purified protein was loaded as a positive control, and the wild-type CL-14 extract was used as a negative control. The band profiles observed on the blots (Figure 2B) indicate that A2 is expressed only in neomycin-resistant parasites transfected with pROCKNeoA2. No expression was detected in the control group, represented by CL-14 WT. The A2 protein expressed by the transgenic CL-14 A2 parasites was further confirmed by immunofluorescence using monoclonal anti-A2 antibodies (Figure 2C). The last result supports the western blot data, whereby CL-14 WT was negative, and only the transfected parasites was positive for the A2 protein.

### CL-14-Based Live Vaccine Induces Potent Immune Responses and Lead to Protection after *L. infantum* Challenge

After the expression of the rA2 by CL-14 A2 parasite had been confirmed, the ability of such vector to elicit specific humoral and cellular immune responses in mice was tested. For this purpose, we immunized mice with a prime/boost homolog protocol with the CL-14 A2, control wild-type CL-14 parasites, and also the rA2 protein along with Alum/CpG (Figure S2 in Supplementary Material). Negative control mice received only PBS.

**Figure 3:**
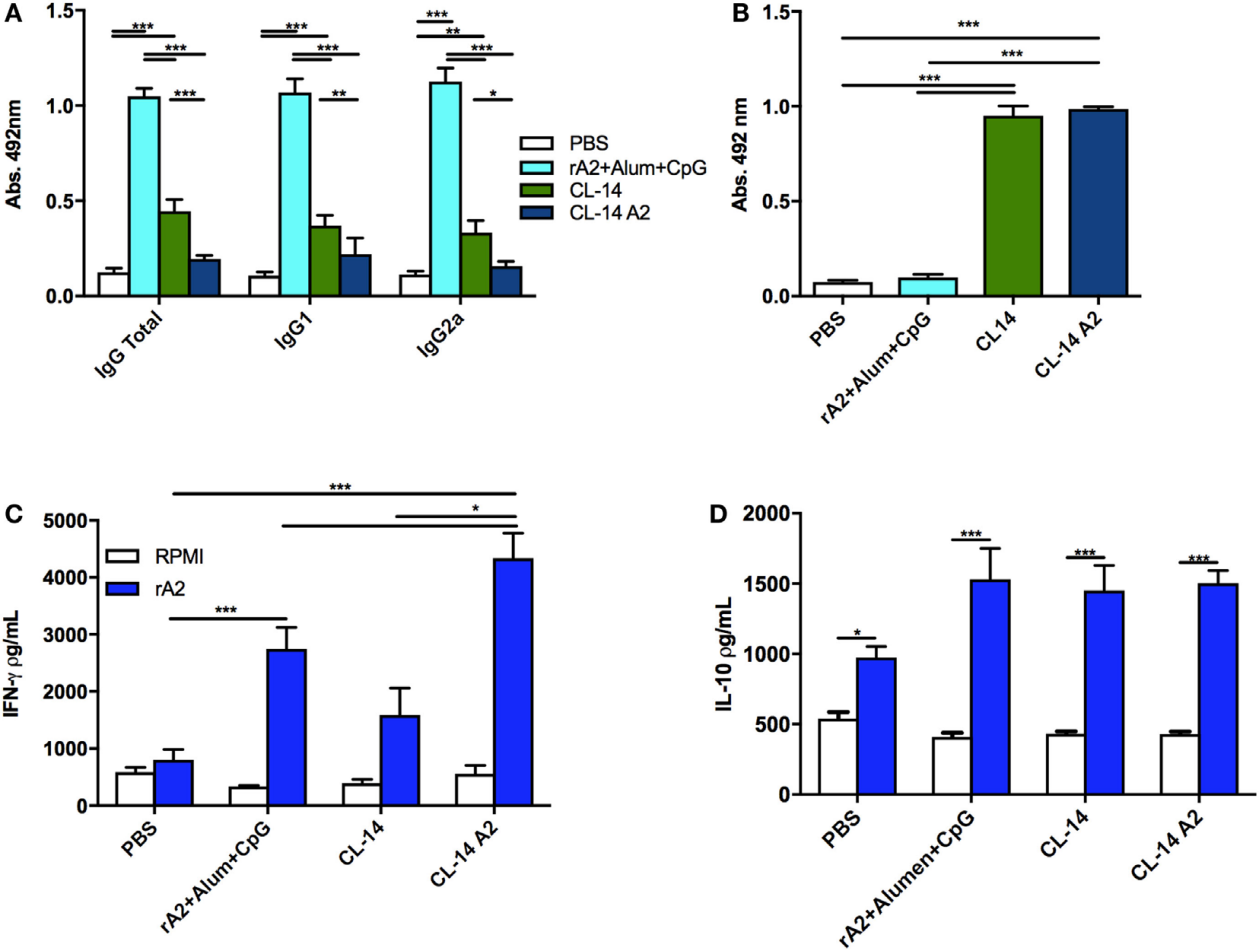
Prime/boost immunization with CL-14 amastigote 2 (A2) induces an effective immune response in immunized BALB/c mice. **(A)** Specific antibody response (total IgG, IgG1, and IgG2a) induced by each immunization protocol was measured 21 days after the last immunization dose using serum samples of immunized mice. **(B)** Levels of total IgG against the live vaccine vector CL-14 were measured by enzyme-linked immunosorbent assay (ELISA) using serum samples of immunized mice. As a coating antigen, 10 µg of total CL-14 antigen extract was used. Mice immunized as previously described were euthanized 21 days after the last immunization dose. Spleen cells were cultured, and supernatants were used for IFN-γ **(C)** or IL-10 **(D)** analyses by ELISA. For *in vitro* re-stimulation, we used either RPMI or rA2. *p < 0.05, **p < 0.01, and ***p < 0.001 analyzed by one-way ANOVA followed by Tukey’s multiple comparison test. Results are expressed as the mean and SEM values of duplicate assays using four to six different animals from each group individually analyzed.

With respect to the anti-rA2 humoral immune responses, mice immunized with rA2 + Alum/CpG showed a higher antibody response (IgG, IgG1, or IgG2a) when compared to mice immunized with the transgenic or control parasites (Figure 3A). Since mice immunized with the parental CL-14 or with the transgenic CL-14 A2 induced similar antibody levels to those observed in the control groups, we performed an ELISA using the total extract of *T. cruzi* CL-14. This assay was designed to confirm that the immunizations using these parasites were efficient. As seen in the Figure 3B, serum samples of animals immunized with the CL-14 parasites (transgenic or otherwise) were able to induce high levels of antibodies to the total extract of the CL-14. The same was not observed in the serum samples of animals immunized with PBS only or the recombinant protein formulations.

Considering that IFN-γ is the main cytokine involved in protection against *Leishmania*, we evaluated its production by spleen cells of mice vaccinated with the different formulations, using an ELISA assay, 21 days after the last immunization dose. When rA2 was used as a stimuli, the animals immunized with the respective recombinant protein associated with Alum/CpG or with the live vaccine (CL-14 A2) were able to produce increased levels of IFN-γ when compared to the control group (Figure 3C). Interestingly, immunization with the wild-type parasite CL-14 was also able to induce significantly increased production of IFN-γ. We also measured the levels of IL-10, an important cytokine related with parasite persistence in the host. Although increased IL-10 levels were detected after rA2 stimulation in all immunized groups, no significant differences were observed among them (Figure 3D).

**Figure 4:**
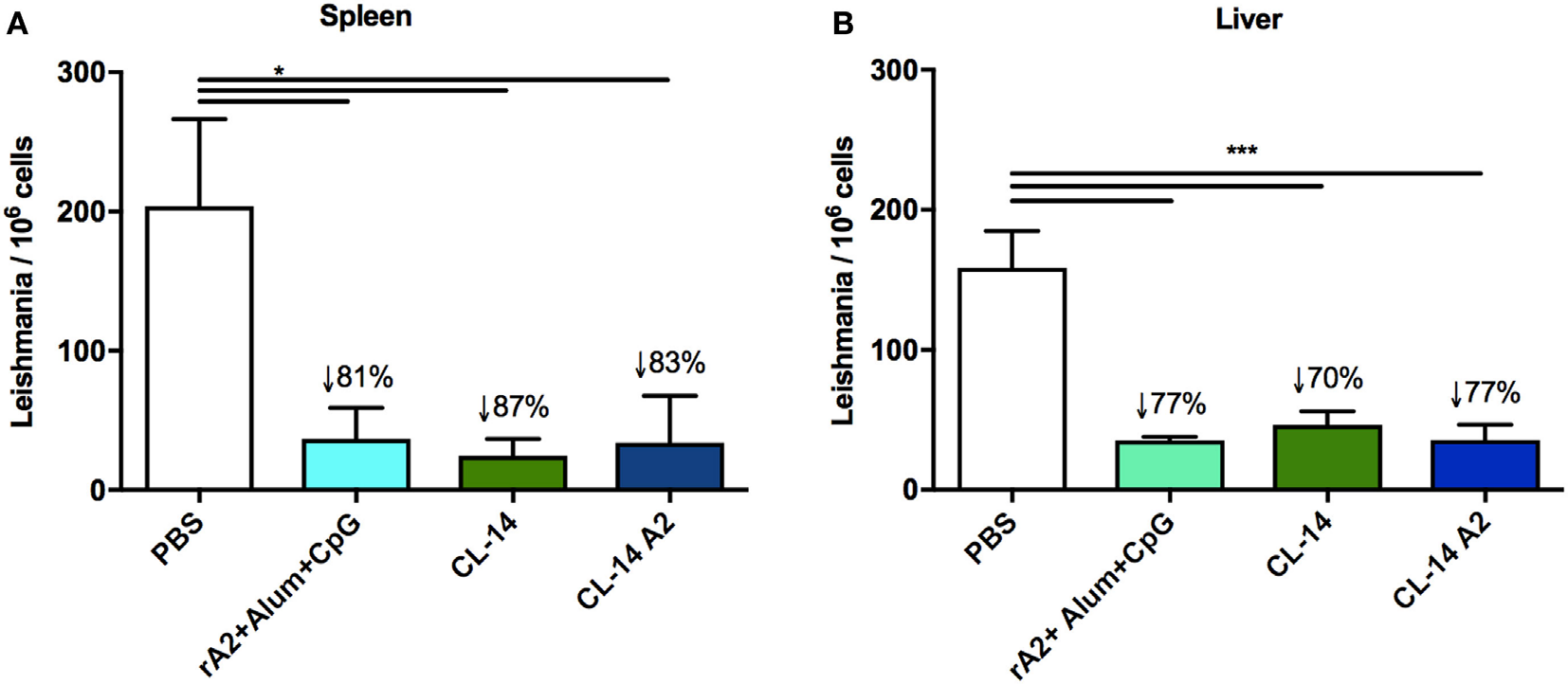
Prime/boost immunization with CL-14 amastigote 2 (A2) induces protection in immunized BALB/c mice 30 days after the challenge animals were euthanized, and DNAs extracted from the spleen **(A)** or liver **(B)** were used for parasite burden using qPCR assay. The reduction percentage of parasite burden, based on the phosphate-buffered saline (PBS) group is shown for each group. *p < 0.05 and ***p < 0.001 analyzed by one-way ANOVA followed by Tukey’s multiple comparison test. Results are expressed as the mean and SEM values of duplicate assays using four to six different animals from each group individually analyzed.

Parasite loads in mice of all groups were determined in the spleen (Figure 4A) or liver (Figure 4B) by quantitative real-time PCR. The results revealed that mice immunized with rA2 + Alum/CpG (78%) as well as those immunized with control CL-14 (71%) and the transgenic parasite expressing A2 (78%) had a significant decrease in parasite burden when compared to the control group (PBS).

### *T. cruzi* Proteins Share Similarity with rA2

Since animals immunized with CL-14 were able to produce elevated levels of IFN-γ when stimulated with rA2, we asked if this protein or its previous predicted epitopes would share sequence similarity with CL-14 proteins. Therefore, a BLASTp analysis was performed to address if the sequence covering most of the described B and T cell epitopes for A2 (SASAEPHKAAVDVGPLSVGPQSVGPLSVGPQAVGPLSV) (11) would have similarity with *T. cuzi* proteins. Table 1 shows the BLASTp results of proteins that had similarities in the restricted epitope sequence.

**Table 1:** Sequence comparison among rA2 and *Trypanosoma cruzi* CL Brener proteins.

*T. cruzi* proteins	Protein identity (%)	Query cover (%)	Similar sequences to A2 mapped epitopes: SASAEPHKAAVDVGPLSVGPQSVGPLSVGPQAVGPLSVGPQ
Transialidases (XP_808957)	32	91	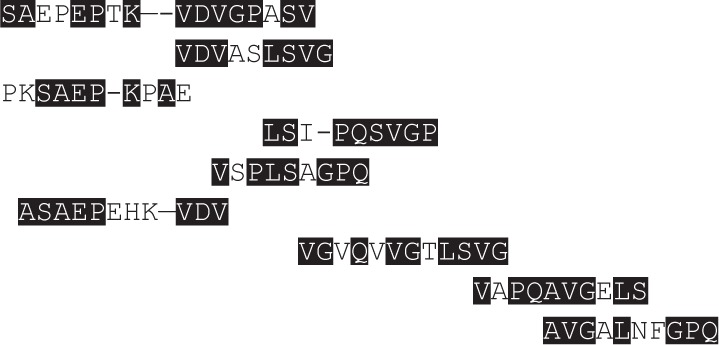
Transialidase (TcCLB.508247.90)	50	11	
Transialidase (XP_808957)	32	91	
Hypothetical protein (TcCLB.506863.70)	80	80	
Hypothetical protein (XP_814399)	50	65	
Mucin TcMucII (TcCLB.510583.70)	77	47	
Amino acid permease (XP_814539.1)	24	54	
Zeta tubulin (XP_817160)	62	76	
Surface protease GP63 (XP_804359.1)	58	70	

## Discussion

To date, the best example of long-lasting protection against leishmaniasis has been achieved through a previous infection with the parasite *L. major* that usually causes local and benign cutaneous lesions, which may spontaneously heal, resulting in the induction of cellular immune responses (28). Based on this, a technique called leishmanization, consisting on the inoculation of infective material from cutaneous wounds, was used in the Middle East, to prevent cutaneous leishmaniasis (29, 30). The caveats of this procedure include safety issues and standardization. However, it indicates that a vaccine is feasible and suggests that chances of success are higher using a live vector strategy (28) although this issue may be eventually circumvented by the use of recombinant protein formulations.

Nonetheless, the development of clinically protective vaccines for VL remains a global challenge (31, 32). The majority of existing vaccines for bacteria and virus are based on the production of specific neutralizing antibodies (33, 34). Conversely, an efficient vaccine for intracellular parasitic infections, such as leishmaniasis, should elicit strong and long-lasting T cell-mediated immunity (35, 36). Therefore, the use of Th1-type adjuvants, including toll-like receptors (TLRs) agonists, as well as systems that prolong antigen exposure and mimic the infection are important strategies to promote and maintain a T cell-based protective response (37, 38).

Among the candidate antigens for second-generation vaccines against VL, A2 is a well-established molecule, employed in several preclinical leishmaniasis prevention protocols and, in combination with saponin, constitute the commercially available veterinary vaccine, Leish-Tec^®^, which is licensed on the Brazilian market since 2008 (13, 14, 27). Although extensively tested in dogs, the Leish-Tec^®^ formulation cannot be directly transposed to human trials, since the adjuvant saponin, which induces lipid bodies and cross-presentation by DCs (39), is not approved for human use. Therefore, progress toward a human VL vaccine may require more specific and safe adjuvants, other than saponin (40). In addition, the original A2 sequence has significant drawbacks for production in industrial settings that may be circumvented. To improve recombinant expression and purification of this antigen, we developed an optimized gene coding for a shorter homolog of the A2 protein, which contains 10 repetitions, instead of the 40–90 repetitions from the original sequence. Codon optimization and the reduction of the repetitive sequences simplified the production process of the recombinant protein (41, 42). In addition, protein expression efficiency and yields were increased. These parameters are especially relevant in the context of quality control and high-scale production at industrial settings. Next, we tested the optimized A2 protein in different vaccine formulations to validate its immunogenicity, when associated with adjuvants more suitable for human use to human use. Our results indicated high levels of protection in BALB/c mice, by combining A2 with MPLA or CpG B297, corroborating previous results that indicate A2 as a promising candidate antigen, including those of preclinical trials in dogs or in Rhesus monkeys, a closer animal model to human VL (43). The combination of A2 with MPLA and ODN was based on prior evidence that these molecules are TLR4 and TLR9 agonists, respectively, with the ability to trigger Th1 responses (44). In addition, both TLRs are activated during *L. donovani* infection (45). Of note is the fact that the protection levels induced by the rA2/CpG B297 formulation against experimental VL were higher than those previously described in BALB/c mice immunized with formulations containing saponin, including the commercial Leish-Tec^®^ vaccine (46). Although CpGs had emerged as good inducers of Th1 immune responses, the combined formulation with Alum increases the antigen/adjuvant depots, leading to an improved uptake by professional presenting cells, strengthening specific immune responses (47). CpG B297 is an ODN present in *T. cruzi* that is associated with enhanced Th1-type adjuvant activity, compared to other standard bacterial CpGs (24). More specifically, the rational selection of CpG B297 was founded on the recognition of this molecule by TLR9 homologs in both mice and human cells (24, 48). In contrast to MPLA, which is a bacterial-derived molecule, or saponin, which is purified from *Quillaja saponaria*, the synthetic nature of CpG B297 may circumvent problems generally associated with other adjuvants, including scale production and the lack of endotoxin or other contaminants, thus improving safety. Therefore, the use of CpG B297 molecule may more easily translate results of preclinical tests to human clinical trials, turning more feasible significant step toward a human vaccine.

The IFN-γ cytokine is the main Th1 marker associated with host protection in VL infection (49, 50). Induction of high levels of antigen-specific IFN-γ was detected for the groups receiving rA2, as well as CL-14 WT and transgenic CL-14 A2 vaccinated groups. Surprisingly, the group immunized with the CL-14 reference strain produced elevated levels of IFN-γ, when stimulated with A2. This intriguing result led us to search for sequence similarities among A2 predicted epitopes and *T. cruzi* proteins. Common epitopes for B cells are known among trypanosomatids and, depending of the transmission areas and the diagnostic test used, may hamper specific diagnosis between *T. cruzi* and *Leishmania* spp infections (51). Therefore, it is plausible that common T cell epitopes might be shared by these parasites, with different degrees of affinities for TCRs. Partial similarity was observed among the sequence SASAEPHKAAVDVGPLSVGPQSVGPV, which contains the A2 B and T cell CD4^+^ and CD8^+^ mapped epitopes (11), and short amino acid sequences present in trans-sialidases, mucin, amino acid permease, GP63, and hypothetical proteins of *T. cruzi*, which may be potential T cell epitopes. Although these findings require further experimental support, they may explain why cells from CL-14 vaccinated animals produced IFN-γ upon rA2 stimulation. Among these proteins, members of the trans-sialidase superfamily are widely studied as antigen candidates for vaccination against Chagas disease since they lead to robust humoral and CD8^+^ T cell-mediated immunity (52–55). In addition to these potential T cell epitopes, the *T. cruzi* CL-14 parasite has several immunomodulators, such as glycoinositolphospholipids and CpG ODNs (24, 56), explaining why the live vaccine possess intrinsic adjuvant properties.

Low levels of antigen-specific antibodies were observed for the CL-14 vaccinated groups. Other vaccine vectors that elicit Th1-biased responses favoring antigen presentation by MHC class I and activation of CD8^+^ T cell, such as adenovirus or plasmid DNA, also lead to low antibody induction depending on the administration protocol (43). In contrast, the vaccination with the rA2 in combination with alum and CpG B297 led to high levels of antigen-specific total IgG, IgG1, and IgG2 antibodies. Generation of antibody-based response was expected for recombinant protein vaccines, as observed in previous studies testing these antigens (9–11, 13, 14, 57). In addition, aluminum salt adjuvants favor antibody production (58). Although questions remain about the mechanism of alum-driven immunomodulation (59), its combination with Th1 type adjuvants can result in both antibody production and increased IFN-γ levels. This pattern was observed in studies with cancer (18) and dengue virus (60) antigens in formulations containing alum plus CpG.

Immunization with CL-14 or recombinant CL-14 A2 parasites elicited a strong Th1-based cell immunity and displayed protection against a *L. infantum* challenge in BALB/C mice, suggesting CL-14 as a potential vaccine candidate for VL. Several factors justify the use of CL-14 as a live vaccine for leishmaniasis: (i) a great number of genes are shared by trypanosomatids; (ii) there is a similar intracellular biological cycle, favoring a CD8 T cell induction; (iii) *T. cruzi* has natural immunostimulatory molecules; and (iv) the long exposure time improves the generation of long-lasting immunity (18, 24, 48, 56). There are also other examples of leishmaniasis vaccine candidates based on non-pathogenic live parasites (28). Among them, *Leishmania tarentolae* is a non-pathogenic species to humans, also of particular interest. This lizard-infecting species is not able to persist long enough in mammalian host to cause infection, even in immunocompromised mice. However, this parasite alone is able to induce DC maturation, elicit Th1-type response, and induce partial cross protection against *L. donovani* challenge (61). Therefore, *L. tarentolae* has been tested as a vaccine vector and recombinant parasite expressing different heterologous antigens, including A2, which led to protection in mice (10, 62–64) and dogs (36). A major concern with live vaccination strategies is related to safety issues. For CL-14, this topic has been addressed many times. In tests with newborn mice, which are very susceptible to Chagas infection, no parasite was found in tissue or blood after inoculation of CL-14 (18, 65). In addition, in experiments with knockout mice, lacking important immunologic mechanisms, such as CD8^+^ T cells, CL-14 was unable to generate disease (7). Regardless these safety issues, successful vaccination studies using live vectors have been also reported for other parasitic diseases such as malaria (66) and toxoplasmosis (67, 68). Noteworthy, the most promising malaria vaccine, which is in advanced clinical trial, is based on i.v. administration of radio-attenuated sporozoites (69). CL-14 was previously reported protective against Chagas disease and cancer, when expressing NY-ESO antigen (18, 24). Our results confirm the potential of live vaccination strategies for VL, specifically with the CL-14 model, increasing its range of target diseases. Nevertheless, even if the translation of this live vaccine candidate as a licensed human vaccine would not be feasible, the reported *T. cruzi* CL-14 data add important contribution to the vaccinology field, showing the potential of a multi-protozoa vaccine, which may favor future development of alternative vaccines for such complex diseases.

Together, our results support the success of the newly designed rA2 vaccine formulations and the *T. cruzi* CL-14 to induce strong T cell-mediated immune responses and protection against VL in animal models. Both vaccination strategies reveal promising alternatives for the development of new vaccine formulations for VL. On one hand, the CL-14 data expand the evidence that live vaccine vectors may be useful to induce cross protective responses and development of multiparasite vaccines; however, the A2 recombinant protein formulation is a more feasible vaccine to progress to human clinical trials.

## Ethics Statement

Mice experiments were approved by and conducted according to animal welfare guidelines of the Ethics Committee of Animal Experimentation from Federal University of Minas Gerais under the approved protocol number 73/2009.

## Author Contributions

CJ and AF conceived the project and designed the experiments. AA, LM, DD, and FN were responsible for developing the experiments, data acquisition, analysis, and interpretation. AA, LM, DD, and CJ wrote the paper. LD was responsible for the rA2 expression optimization. RG made significant technical and conceptual contributions to the manuscript and data interpretation. All the authors provided intellectual content and approved the final version of the paper.

## Conflict of Interest Statement

The author CJ, AF, RG, LD, and DD are holders of two patent deposit related to the present work. All other authors declare that the research was conducted in the absence of any commercial or financial relationships that could be construed as a potential conflict of interest.
