# Supplementary material for: New Vaccine Formulations Containing a Modified Version of the Amastigote 2 Antigen and the Non-Virulent Trypanosoma cruzi CL-14 Strain Are Highly Antigenic and Protective against Leishmania infantum Challenge
Source: Front Immunol. 2018 Mar 15;9:465. doi: 10.3389/fimmu.2018.00465 (PMC5863692; doi:10.3389/fimmu.2018.00465)
Supplement: Supplementary file 1 [file Data_Sheet_1.PDF]

Supplementary Material

New vaccine formulations containing a modified version of the A2 antigen and the non-virulent *Trypanosoma cruzi* CL-14 strain are highly antigenic and protective against *Leishmania infantum* challenge

Ana Paula M. M. Almeida; Leopoldo F. M. Machado; Daniel Doro; Frederico C. Nascimento; Leonardo Damasceno; Ricardo Tostes Gazzinelli; Ana Paula Fernandes*; Caroline Junqueira*

* **Correspondence:** Ana Paula Fernandes: apfernandes.ufmg@gmail.com and Caroline Junqueira: carolinejunqueira@cpqrr.fiocruz.br

Supplementary Figure 1.

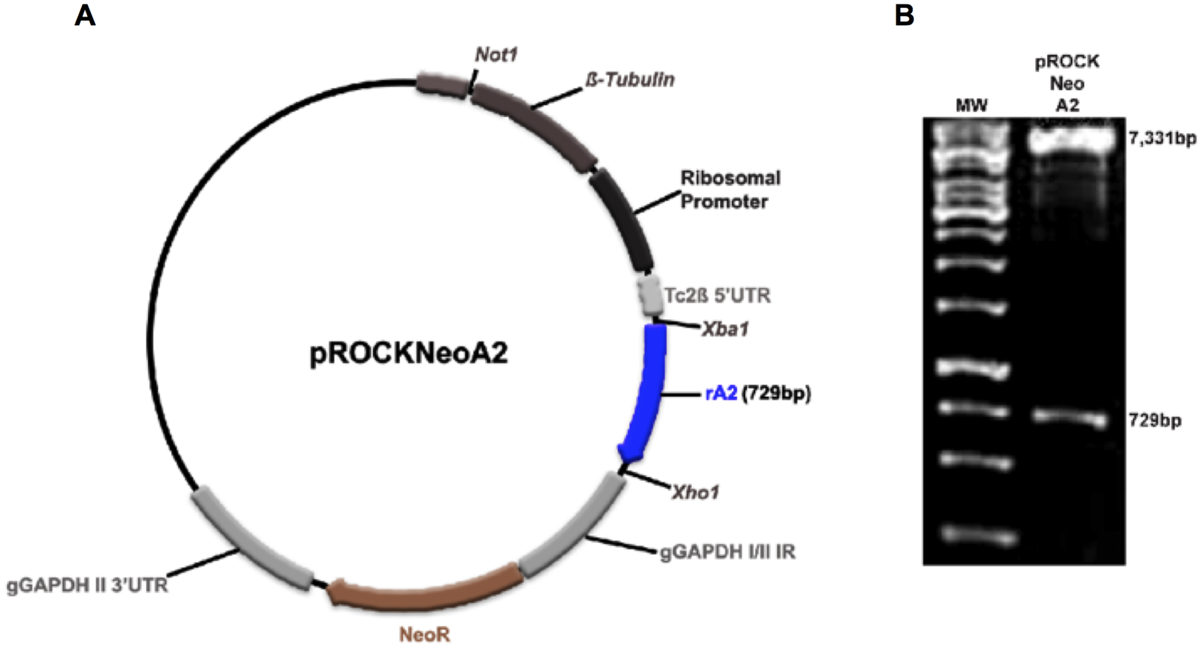

S1. Overview of pROCKNeoA2 structure and construction. **(A)** Schematic representation of the integrative plasmid pROCKNeoA2 showing the enzyme restrictions sites used, integrative site, antibiotic resistance and transgene position. **(B)** Agarose gel electrophoresis of cleavage products of pROCKNeoA2 with *Xba*I and *Xho*I resulting in the expected fragment sizes of 726 pb (A2 sequence) and 7331 pb (pROCKNeo).

Supplementary Figure 2.

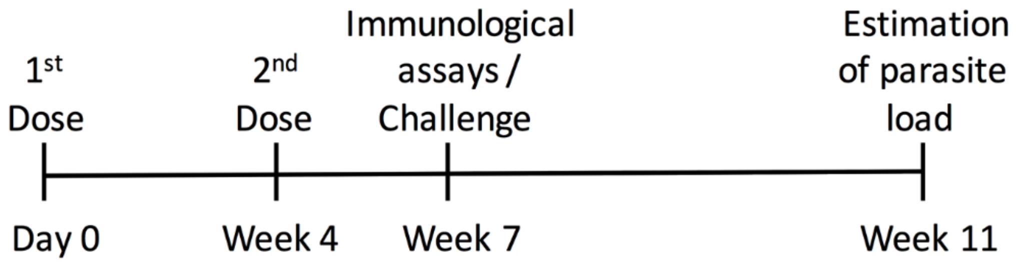

Figure S2. Schematic representation of the imunization schedule. At day 0 and week 4, the antigens were administered as previously described. At week 7, four animals of each group were sacrificed and had their blood and spleen collected for imunological analysis. The remaining 6 animals were challenged, into the right hind footpad, with 1×10^7 stationary phase promastigotes of *L. infantum*. At week 11, all the infected animals were sacrificed and had their spleen and liver collected for parasite burden determination.
